# Comparative phenotypic and genomic analysis of the methanogen Methanomethylovorans thermophila L2FAW and its phylogenomic placement within the Genome Taxonomy Database

**DOI:** 10.1099/acmi.0.001123.v4

**Published:** 2026-05-21

**Authors:** Mathias Wunderer, Andja Mullaymeri, Andreas O. Wagner, Eva Maria Prem

**Affiliations:** 1 Department of Microbiology, Universität Innsbruck, Technikerstrasse 25d, 6020 Innsbruck, Austria

**Keywords:** hybrid genome assembly, Illumina shotgun sequencing, methanogenic archaea, Oxford Nanopore sequencing

## Abstract

The genome of the methylotrophic methanogen *Methanomethylovorans thermophila* L2FAW is not included in the Genome Taxonomy Database (GTDB) so far, even though the strain was first described in 2005. To evaluate its genomic characteristics and placement in the GTDB, we sequenced the genome of *M. thermophila* L2FAW via *Illumina* shotgun and Oxford *Nanopore* sequencing and subsequently did hybrid assembly. The assembled genome consists of 2.25 Mbp (contigs ≥500 bp) with a G+C content of 40 mol%. The quality of the genome is good, which is already apparent from the low L50 (=1) and L90 (=2) metrics. Our assembled genome was highly similar to the metagenome-assembled genome *Methanomethylovorans sp014361205* (GCA_014361205.1_ASM1436120v1_genomic) with an average nucleotide identity of 99.9%. Even though KEGG Mapper Reconstruction results revealed that *M. thermophila* L2FAW harbours all the enzymes necessary for acetoclastic and hydrogenotrophic methanogenesis and *gapseq* predicted formate as a potential substrate for *M. thermophila* L2FAW, no metabolic activity could be observed on acetate, H_2_-CO_2_ (80:20 vol/vol, 2,000 mbar) and on a mixture of H_2_-CO_2_ and formate in lab tests; thus, the obligate methylotrophic lifestyle of the phenotype was confirmed.

## Data Summary

This whole-genome shotgun project has been deposited at the National Center for Biotechnology Information (NCBI) under the BioProject ID: PRJNA1271748. The BioSample accession no. is SAMN48886148, and the two Sequence Read Archive (SRA) accession nos. are SRX29517605 and SRX29517606.

## Introduction

The methanogenic archaeon *Methanomethylovorans thermophila* L2FAW was isolated from a laboratory-scale thermophilic upflow anaerobic sludge blanket reactor and described in 2005 [1]. It is phylogenetically closely related to *Methanomethylovorans hollandica* DMS1 (98% similarity for the 16S rRNA gene) and represented a novel species in the genus *Methanomethylovorans* [1]. The genus name was proposed in 1999 after the isolation of the type strain *M. hollandica* DMS1 from the sediment of an eutrophic freshwater pond in the Netherlands [2]. *Methanomethylovorans* species are obligate methylotrophs, utilizing methanol, methylated amines, methylated sulphides and tetramethylammonium hydroxide [1,3]. As elaborated with the S*ingleM* software suite implemented in *Sandpiper* [4], Methanomethylovorans sp. was also found in other habitats, such as wetlands, biofilms, wastewater, anaerobic digestion plants, soils or mine drainage with relative abundances of up to 30% in, e.g. wetlands (https://sandpiper.qut.edu.au/taxonomy/g__Methanomethylovorans). Coastal wetlands play an integral role in the global carbon cycle, potentially serving as methane sources [5]. In those habitats, methylotrophic methanogenesis is a key metabolic process [6]. In river sediments, *Methanomethylovorans* sp. dominate dimethyl sulphide degradation [7], thereby further contributing to global climate dynamics.

The Genome Taxonomy Database (GTDB) follows a phylogenomic approach based on a set of conserved proteins (120 for bacteria and 53 for archaea), primarily related to ribosomes (https://gtdb.ecogenomic.org/). It defines higher-rank taxa and species via relative evolutionary divergence and average nucleotide identity (ANI), respectively. Moreover, the National Center for Biotechnology Information (NCBI) Assembly Database functions as a genome repository [8]. The genome of *M. thermophila* L2FAW has not been fully sequenced so far, and it is, therefore, not included in the GTDB. Therefore, this study aimed to sequence the whole genome of *M. thermophila* L2FAW, to compare the genome to the phenotype regarding substrate spectrum and to phylogenomically place the strain within the GTDB to possibly provide information on assigned metagenome-assembled genomes of *Methanomethylovorans* with placeholder names, as well as to gain insights into the geographic distribution of *M. thermophila* L2FAW.

## Methods

### Cultivation

The methanogenic archaeon *M. thermophila* L2FAW (DSM 17232) was obtained from DSMZ-Deutsche Sammlung von Mikroorganismen und Zellkulturen (Braunschweig, Germany). The cultivation occurred in a 120-ml serum flask, filled with 50 ml of DSMZ 684 medium with varying carbon sources: 2 ml l^−1^ methanol, 2.5 g l^−1^ sodium acetate, 2,000 (±25) mbar H_2_-CO_2_ (80:20 vol/vol) and 2 g l^−1^ sodium formate with 2,000 (±25) mbar H_2_-CO_2_ (80:20 vol/vol). The incubation temperature was 55 °C, and 10% (vol/vol) cell suspension was used for inoculation. Media preparation under anoxic conditions occurred according to Wagner *et al.* [9].

### Analysis of gas production

Overpressure in the serum flask was measured with a G1113-UT-GE manometer (Greisinger electronic, Germany) using the current atmospheric pressure as reference [Bundesanstalt für Geologie, Geophysik, Klimatologie und Meteorologie (GeoSphere, Austria) (https://www.geosphere.at/de)]. The biogas composition (H_2_, CH_4_, CO_2_) in the headspace of the serum flask was determined using a GC 2010 gas chromatograph (Shimadzu, Japan) according to Wagner *et al.* [10]. The gas chromatograph was calibrated before each measurement with a reference gas from Messer (Austria) containing 60% CH_4_, 30% CO_2_ and 5% H_2_. CH_4_ production was calculated according to Wunderer *et al.* [11].

### DNA purification

DNA purification was carried out with the Quick-DNA^™^ HMW MagBead Kit (Zymo Research, USA) according to the manufacturer’s recommendations. The DNA was quantified with Quant-iT^™^ PicoGreen^™^ dsDNA Assay Kit (Invitrogen, Germany), resulting in a concentration of 14.1 ng µl^−1^. Additionally, the DNA quality was checked with a NanoDrop^™^ 2000 c (Thermo Scientific^™^) spectrophotometer (260/280=1.82 and 260/230=2.00).

### Next- and third-generation sequencing

*Illumina* shotgun next-generation sequencing was done on a NovaSeq device by Novogene (Cambridge, UK). Third-generation sequencing was done with a MinION Mk1B device (Oxford *Nanopore* Technologies, Great Britain) in-house using the MinKnow software v24.06.5 (Oxford *Nanopore* Technologies, Great Britain), an R10.4.1 MinION Flow Cell (FLO-MIN114, Oxford *Nanopore* Technologies, Great Britain), the Ligation Sequencing Kit V14 (SQK-LSK114, Oxford *Nanopore* Technologies, Great Britain) and companion products (NEB, USA) according to the manufacturer’s recommendation.

### Processing of the raw reads and genome assembly

Base calling of the *Nanopore* reads occurred with *Dorado* basecaller (0.7.3-linux-x64), using the automatically downloaded HAC model (https://github.com/nanoporetech/dorado). The base called reads were single-read error corrected with the HERRO algorithm (https://github.com/nanoporetech/dorado). We used *Filtlong* (https://github.com/rrwick/Filtlong) without an external reference for quality filtering of the *Nanopore* reads and removed sequences shorter than 1 kbp, as well as the worst 10% (measured by bp, not by read count). Quality filtering and adapter removal for *Illumina* shotgun reads were done with *Trimmomatic* (https://github.com/usadellab/Trimmomatic). Hybrid assembly using *Nanopore* and *Illumina* reads took place with *SPAdes* 4.0.0 (https://github.com/ablab/spades) [12,15]. The completeness of the assembled genome was verified with *Bowtie2* (https://github.com/BenLangmead/bowtie2). *SAMtools* (https://github.com/samtools/samtools?tab=readme-ov-file) was used to sort and index the assembled genome and remove unpaired reads [16]. Polishing of the genome occurred with *Pilon* (https://github.com/broadinstitute/pilon?tab=readme-ov-file) and the quality of the assembled genome was evaluated with *QUAST* 5.3.0 (https://github.com/ablab/quast) [17].

### Annotation, taxonomic classification and metabolic reconstruction

**Fig. 1.**
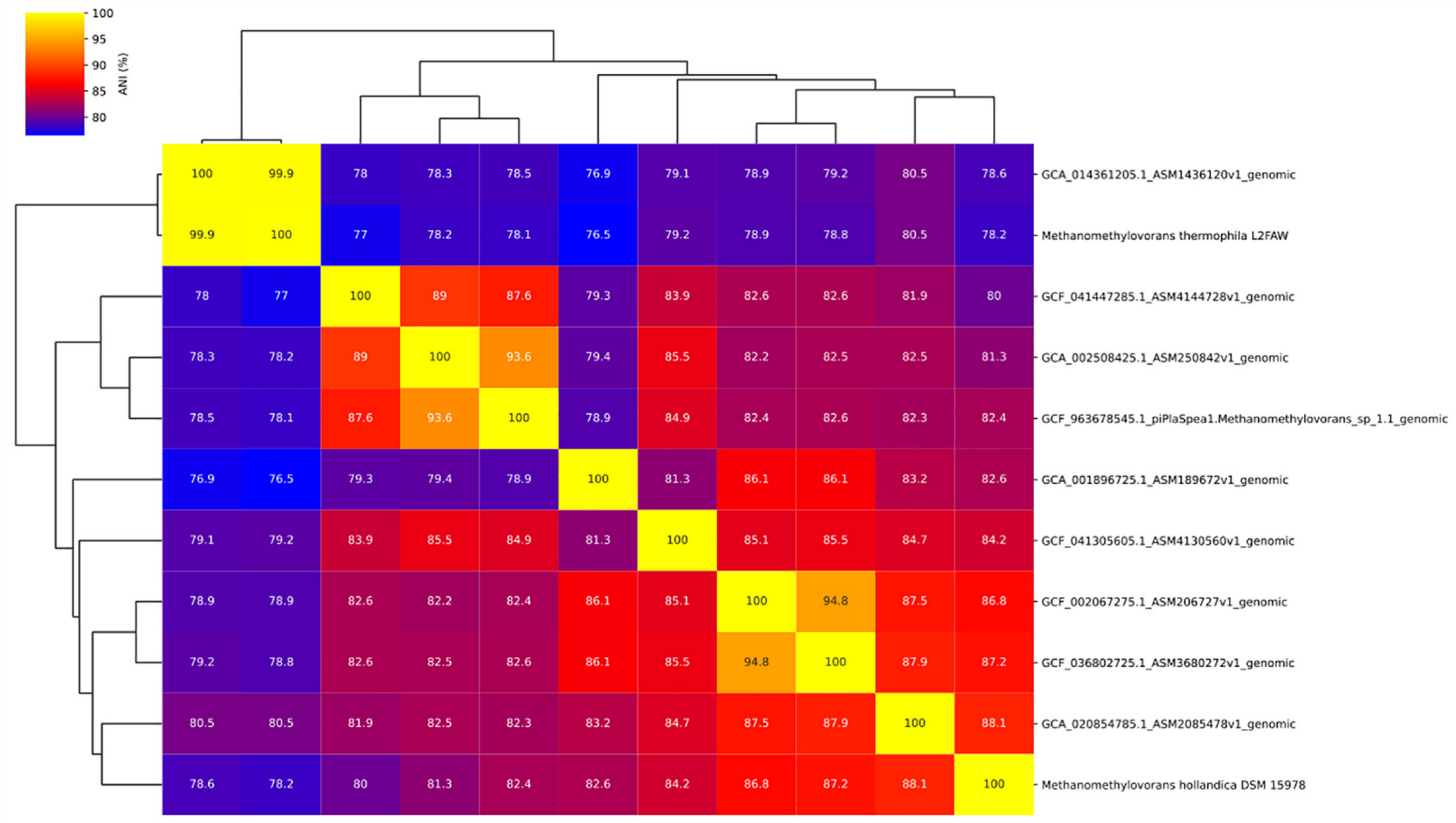
Comparison of ANI values of the GTDB species representatives of *Methanomethylovorans* sp. with our assembled genome (*M. thermophila* L2FAW). The completeness, align fraction reference and align fraction query of the GTDB species representatives are shown in Table S5.

We downloaded GTDB species representatives of *Methanomethylovorans* sp. (*n*=10) from the NCBI [18] in June 2025 (GTDB release r226). GTDB representatives are non-redundant, curated biological genomes of high quality. Together with our assembled genome of *M. thermophila* L2FAW (*n*=11), ANI values were calculated in a multifactorial manner via *ANIclustermap v2.0.1* using *skani* as calculation tool [19] (https://github.com/moshi4/ANIclustermap) and visualized with the options *--annotation, --fig_width 18, --cmap_gamma 0.8, --cmap_colors, --mode skani* (Fig. 1). For information on the aligned fraction (AF), we used *skani v0.2.2* with following options: --slow, --min-af 10 and --robust [20]. Furthermore, we downloaded all GTDB species representatives of the family *Methanosarcinaceae* (*n*=139) from the NCBI [21] in February 2026 (GTDB release r226). The 53 marker proteins for archaea were identified and aligned with *GTDB-Tk* (v2.4.1; *GTDB-Tk* reference data version r226) (https://github.com/Ecogenomics/GTDBTk) [22]. The alignment of these 140 genomes (139 GTDB species representatives and our assembled genome) was used for the phylogenomic reconstruction with *iqtree v3.0.1* [23] with the options: -m MFP [24], -bb 1000 [25] and alrt 1000 (https://github.com/iqtree/iqtree3). The model finder selected Q.PLANT+F+R7 as the best-fit model according to the Bayesian information criteria. The phylogenomic tree was visualized with the online display tool *iTOL* (https://itol.embl.de) [26].

The completeness and the contamination of the genome were determined using *CheckM2 1.1.0* (https://github.com/chklovski/CheckM2) [27]*. Prokka* v1.14.5 (https://github.com/tseemann/prokka) [28] was used for the annotation of the genome. The resulting amino acid sequences were processed with *BlastKOALA* (with BlastKEGG Orthology Ank Links Annotation) (https://www.kegg.jp/blastkoala/) [29] and visualized with *KEGG Mapper Reconstruction Result* [30]. Additionally, prediction of metabolic pathways, transporter inference, metabolic model construction and multi-step gap filling took place with *gapseq* (https://github.com/jotech/gapseq) [31,32]. The received metabolic model was further processed in *RStudio* 2024.12.1+563 with the R version R-4.4.2 (Posit PBC, USA), using the packages *data.table* [33] and *sybil* [34] (https://gapseq.readthedocs.io/en/latest/tutorials/archaea.html) [35]. S*ingleM* software suite (https://github.com/wwood/singlem) implemented in *Sandpiper* (https://github.com/wwood/sandpiper) [4] was used to determine the distribution of the metagenome-assembled genome (MAG) *Methanomethylovorans* sp014361205 and *Methanomethylovorans* sp. in general.

## Results and discussion

### Genome quality assessment

The quality of the assembled genome and its closest match (MAG GCA 014361205.1) is shown in Table S1, available in the online Supplementary Material. In total, 53 contigs were created, of which 3 were longer than 500 bp. The genome comprises 2.27 Mbp (2.25 Mbp contigs ≥500 bp) with a G+C content of 40 mol%. According to *CheckM2*, the genome has a completeness of 98.97% and contamination of 0.57%. This is comparable with other in the GTDB listed *Methanomethylovorans* genomes, i.e. 2.15 Mbp for the MAG GCA_014361205.1 (completeness 99.67%) (https://gtdb.ecogenomic.org/genome?gid=GCA_014361205.1) or 2.71 Mbp for the type species of the genus *Methanomethylovorans*, *M. hollandica* GCF_0000328665.1 (completeness 99.84%) (https://gtdb.ecogenomic.org/genome?gid=GCF_000328665.1). The G+C content is slightly higher than determined by Jiang et al. [1] (37.6 mol%) and is comparable with that of other *Methanomethylovorans* sp. (34.4 mol% of *M. hollandic*a DSM1 and 39.2 mol% of *Methanomethylovorans uponensis*) [1,36]. N50 and N90 lengths were 1.56 Mb and 685 kb, respectively. The minimum number of contigs needed to cover 50 and 90% of the assembled genome was 1 and 2, respectively (L50=1, L90=2). 99.96% of the reads are mapped with a coverage of 100% (1×), an average coverage depth of 414 and zero uncalled bases per 100 kb. These metrics indicate that hybrid assembly worked well, and the genome is of good quality.

### Taxonomic evaluation

The investigated genome of *M. thermophila* L2FAW had the highest similarity with the MAG *Methanomethylovorans sp014361205* (GCA_014361205.1_ASM1436120v1_genomic), with a genome ANI of 99.9% (Fig. 1). Genomes with an ANI ≥95% (or ≥97%) are usually clustered as the same species [37]. Moreover, 96.5% of the reference and 92.2% of the query were covered by the alignment (AF value) (Table S5). So far, MAG *Methanomethylovorans sp014361205* was found globally in bioreactor sludge, soil, wastewater or oil production facilities with relative abundances below 5% (https://sandpiper.qut.edu.au/taxonomy/s__Methanomethylovorans%20sp014361205). This is in accordance with the isolation source of *M. thermophila* L2FAW.

All other *Methanomethylovorans* sp. genomes showed ANI and AF values ≤80 and <40%, respectively, when compared with * M. thermophila* L2FAW (Table S5). This indicates a high genomic distance between *M. thermophila* L2FAW and those *Methanomethylovorans* sp. genomes.

**Fig. 2.**
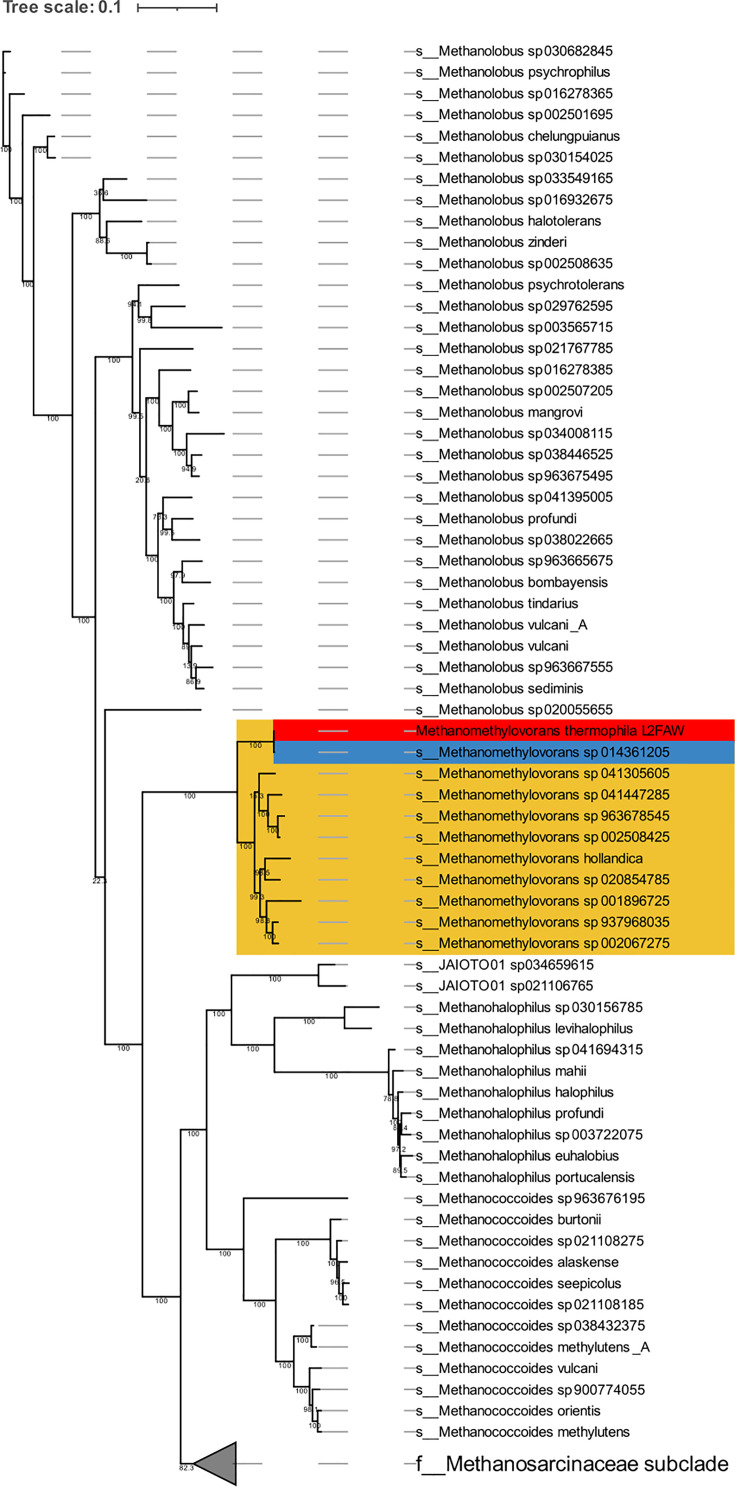
Phylogenomic tree based on the 53 archaeal marker proteins of all available GTDB species representatives (*n*=139) and our assembled genome *M. thermophila* L2FAW. In red, our assembled genome; in blue, the MAG with the highest similarity to our genome (ANI=99.9%); and in yellow, all members of the genus *Methanomethylovorans*. The given numbers at the branches reflect bootstrap values.

According to Parks *et al*. [37], intragenus species within genera with multiple species show a distribution of ANI values between 78 and 95%. Since all ANI values except for the MAG *Methanomethylovorans* sp014361205 are between 76.52 and 80.38% (Fig. 1), when compared with our assembled genome, we calculated a phylogenomic tree with all available GTDB species representatives of the family *Methanosarcinaceae* (Fig. 2). Our assembled genome (in red) clusters together with the MAG *Methanomethylovorans* sp014361205 (in blue) in the same clade as the sister group of all the other *Methanomethylovorans* sp. (in yellow). This shows us that it belongs to the genus *Methanomethylovorans*, even when the ANI values in the pairwise comparison are mostly <80% (for the entire tree, please see Fig. S1).

### Genome annotation and metabolic prediction

**Fig. 3.**
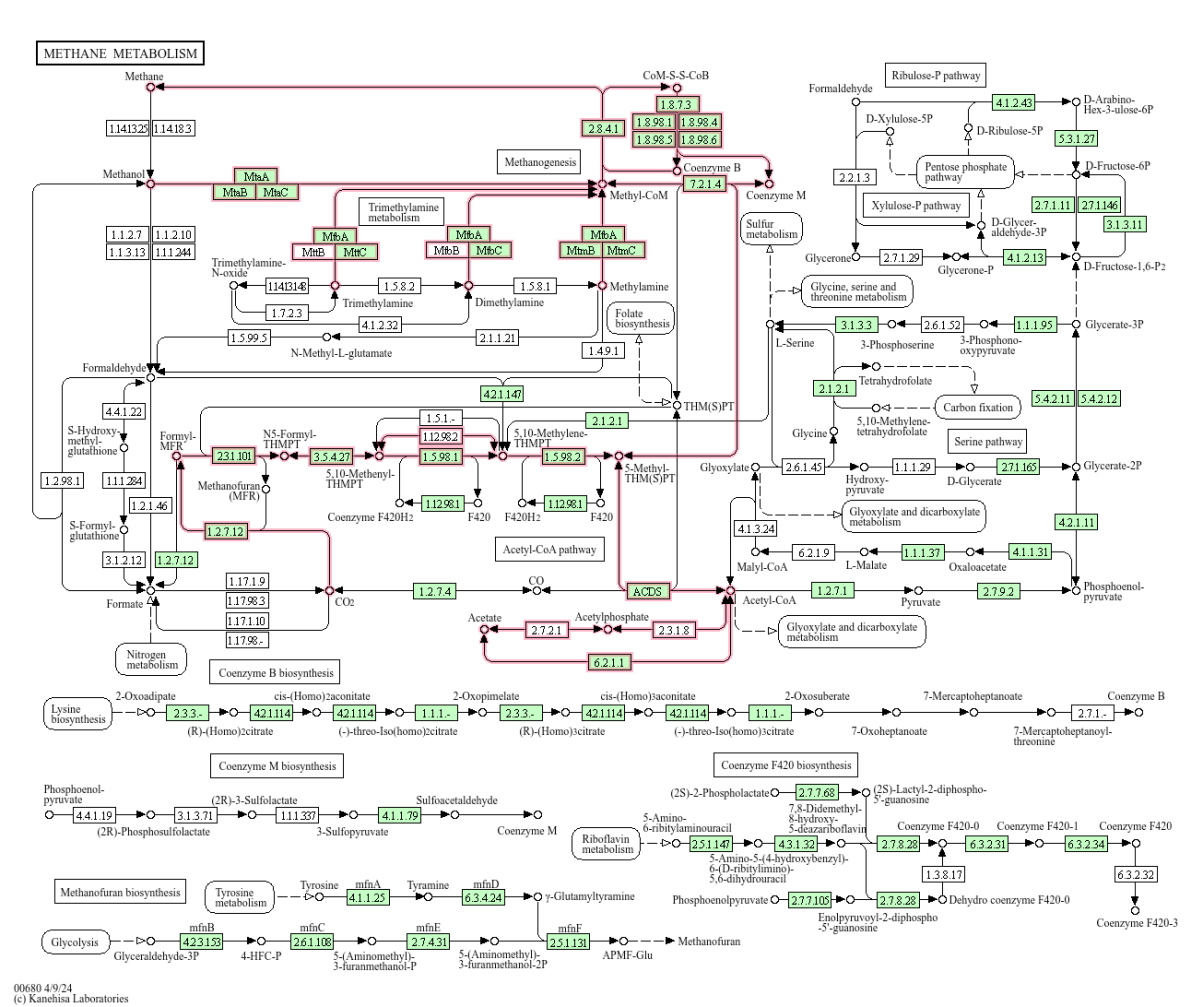
Methane metabolism map created with BlastKOALA and KEGG Mapper Reconstruction Result. In green are the enzymes that are present in the genome of *M. thermophila* L2FAW. Red lines show the methanogenic pathways: M00567 (CO_2_ ->methane, complete 8/8), M00357 (acetate ->methane, complete 5/5), M00356 (methanol ->methane, complete 3/3) and M00563 (mono-, di- and trimethylamine ->methane, complete 4/4).

According to *Prokka*, the genome has 2,168 CDS, of which 1,107 could not be assigned and classified as hypothetical proteins (Table S2). The genome encodes two 5S rRNAs, three 16S rRNAs, two 23S rRNAs, 3 repeated regions (CRISPR) and 44 tRNAs. MAG GCA_014361205.1 contains slightly fewer CDS with 2,139 and 47 tRNAs (Table S2) (https://gtdb.ecogenomic.org/genome?gid=GCA_014361205.1). For comparison, the genome *Methanomethylovorans holandica* GCF_0000328665.1 contains 2,636 CDS and 52 tRNA (https://gtdb.ecogenomic.org/genome?gid=GCF_000328665.1). The amino acid annotation with *BlastKOALA* and subsequent visualization of the results with *KEGG Mapper Reconstruction Result* revealed that *M. thermophila* harbours all the enzymes that are necessary for acetoclastic and hydrogenotrophic methanogenesis, besides methylotrophic methanogenesis from methanol, mono-, di- and trimethylamine (Fig. 3). The predicted metabolic model (Tables S3 and S4) also showed that formate is a potential substrate for *M. thermophila* and indicates its potential for hydrogenotrophic methanogenesis.

**Fig. 4.**
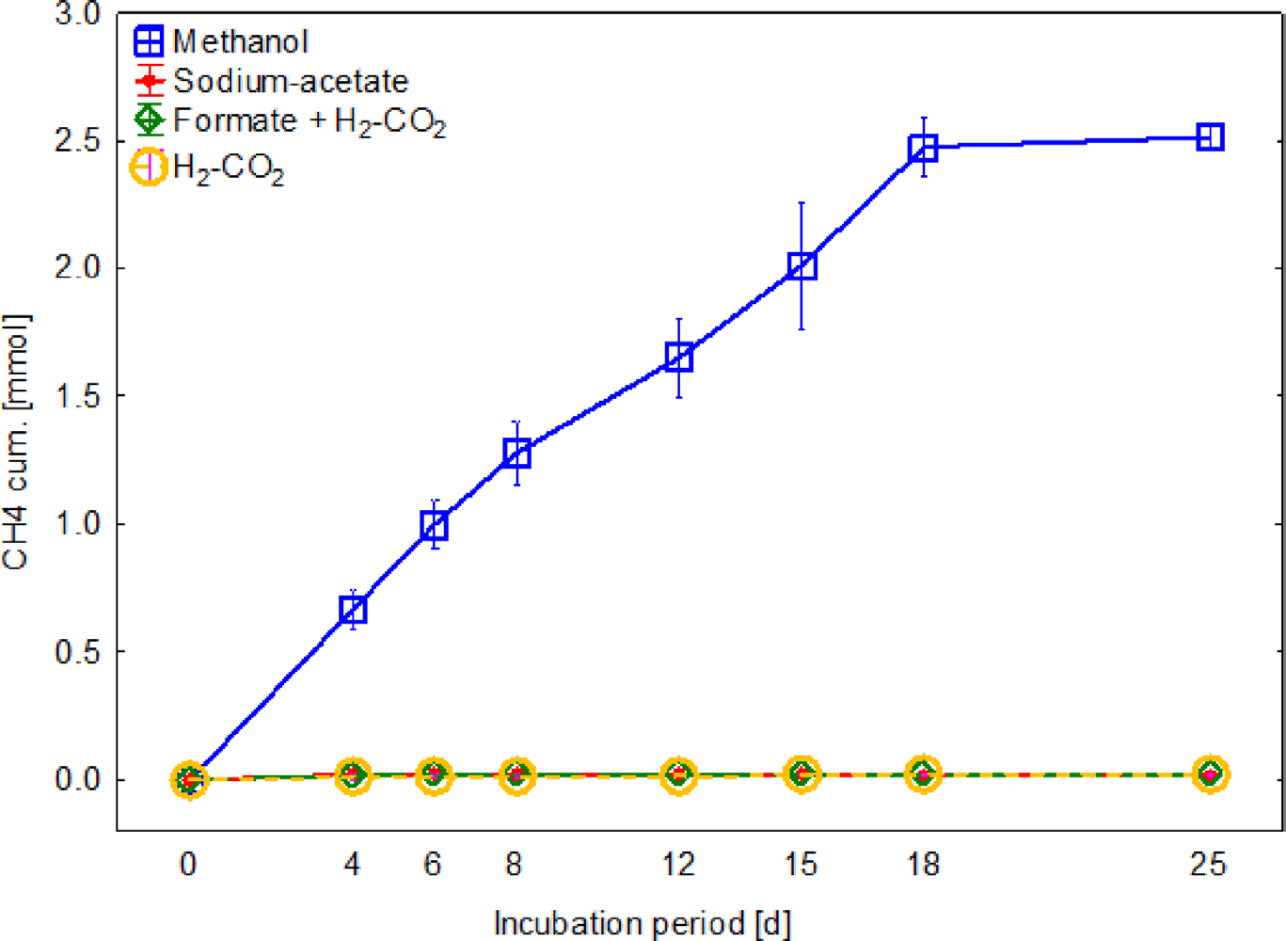
Cumulative methane production of *M. thermophila* L2FAW on various carbon sources: methanol, sodium acetate, H_2_-CO_2_ and a mixture of H_2_-CO_2_ and sodium formate.

However, even if all enzymes required for acetoclastic and hydrogenotrophic methanogenesis are present, no growth could be observed on H_2_-CO_2_ (80:20 2,000 mbar), a mixture of formate and H_2_-CO_2_ (80:20 vol/vol, 2,000 mbar) and acetate in lab tests (Fig. 4). These results confirm the findings of Jiang *et al.* [1], who could demonstrate that *M. thermophila* can only grow on methanol, mono-, di- and trimethylamine but not on H_2_-CO_2_ (80:20 vol/vol, 1,700 mbar), formate or acetate as sole carbon and energy source.

## Supplementary material

10.1099/acmi.0.001123.v4 Uncited Supplementary Material 1.

## Abbreviations

AF: aligned fraction
ANI: average nucleotide identity
GTDB: Genome Taxonomy Database
MAG: metagenome-assembled genome
NCBI: National Center for Biotechnology Information
