# Supplementary material for: Comparative phenotypic and genomic analysis of the methanogen Methanomethylovorans thermophila L2FAW and its phylogenomic placement within the Genome Taxonomy Database
Source: Access Microbiol. 2026 May 21;8(5):001123.v4. doi: 10.1099/acmi.0.001123.v4 (PMC13193254; doi:10.1099/acmi.0.001123.v4)
Supplement: Uncited Supplementary Material 1. [file acmi-8-01123-s001.pdf]

Table S1: Quality assessment of the assembled genome of Methanomethylovorans thermophila L2FAW; evaluated with QUAST 5.3.0 and compared with the closest match MAG GCA 014361205.1 (: data derived from GTDB (https://gtdb.ecogenomic.org/genome?gid=GCA_014361205.1)).*

Parameters	Methanomethylovorans thermophila L2FAW	MAG GCA 014361205.1 (closest match) *
Contigs (>= 500 bp)	53 (3)	170
Total length (contigs >= 0 bp)	2266898	2148332
Total length (contigs >= 500 bp)	2248139	Not available
GC (%)	37.97	38.12
N50	1562897	20519
N90	684500	Not available
L50	1	Not available
L90	2	Not available
Mapped (%)	99.96	Not available
Avg. coverage depth	414	Not available
Coverage >= 1x (%)	100.0	Not available
N's per 100 kbp	0.0	0.0

Table S2: Main results of the genome annotation with Prokka of the assembled genome Methanomethylovorans thermophila L2FAW compared with the closest match MAG GCA 014361205.1 (: data derived from GTDB (https://gtdb.ecogenomic.org/genome?gid=GCA_014361205.1))*

Parameters	Methanomethylovorans thermophila L2FAW	MAG GCA 014361205.1 (closest match) *
Bases	2266898	2148332
CDS (hypothetical proteins)	2168 (1107)	2139
rRNA (5S-16S-23S)	2-3-2	2-1-1
Repeat regions (CRISPR)	3	Not available
tRNA	44	47

Table S3: Potential substrates of Methanomethylovorans thermophila L2FAW according to the predicted metabolic model; created with gapseq and modified in RStudio with the packages data.table and sybil. Ex = compound identification number, met = metabolite, flux = turnover rate mmol/gDW/h.

**ex**	**met**	**flux**
EX_cpd00116_e0	Methanol-e0	-1.310285e+01
EX_cpd00027_e0	D-Glucose-e0	-5.000000e+00
EX_cpd00009_e0	Phosphate-e0	-1.797608e+00
EX_cpd00528_e0	N_2-e0	-8.579085e-01
EX_cpd00047_e0	Formate-e0	-2.881119e-01
EX_cpd00156_e0	L-Valine-e0	-1.000000e-01
EX_cpd00161_e0	L-Threonine-e0	-1.000000e-01
EX_cpd00084_e0	L-Cysteine-e0	-1.000000e-01
EX_cpd00054_e0	L-Serine-e0	-1.000000e-01
EX_cpd00107_e0	L-Leucine	-1.000000e-01
EX_cpd00039_e0	L-Lysine-e0	-1.000000e-01
EX_cpd00322_e0	L-Isoleucine	-8.354430e-02
EX_cpd00066_e0	L-Phenylalanine	-5.443038e-02
EX_cpd00065_e0	L-Tryptophan	-1.683544e-02
EX_cpd00048_e0	Sulfate	-4.314863e-03
EX_cpd00149_e0	Co2+-e0	-9.844590e-04
EX_cpd00244_e0	Ni2+-e0	-4.189187e-04
EX_cpd00220_e0	Riboflavin	-2.273876e-14

Table S4: Potential products of Methanomethylovorans thermophila L2FAW according to the predicted metabolic model; created with gapseq and modified in RStudio with the packages data.table and sybil. Ex = compound identification number, met = metabolite, flux = turnover rate mmol/gDW/h.

**ex**	**met**	**flux**
EX_cpd00180_e0	Oxalate-e0	1.780405e-04
EX_cpd00363_e0	Ethanol-e0	1.221148e-02
EX_cpd00141_e0	Propionate-e0	4.118929e-02
EX_cpd00239_e0	H_2S-e0	4.288029e-02
EX_cpd00055_e0	Formaldehyde-e0	2.117281e-01
EX_cpd11416_c0	Biomass	2.232855e-01
EX_cpd00067_e0	H+-e0	7.335886e-01
EX_cpd00012_e0	PPi-e0	7.901986e-01
EX_cpd00029_e0	Acetate-e0	2.075293e+00
EX_cpd00001_e0	H_2O-e0	1.093367e+01
EX_cpd00011_e0	CO_2-e0	1.412341e+01
EX_cpd01024_e0	Methane-e0	1.953726e+01

Table S5: Average nucleotide identity (ANI), align fraction reference, and align fraction query values of the GTDB species representatives of Methanomethylovorans sp. (GTDB release r226) in comparison with our assembled genome and the respective completeness of these genomes (marks data derived from GTDB (https://gtdb.ecogenomic.org/searches?s=al&q=methanomethylovorans).*

**Name**	**ANI**	**Align fraction reference**	**Align fraction query**	**Completeness [%] ***
GCA_014361205.1_ASM1436120v1_genomic	99.92	96.52	92.23	99.67
GCA_020854785.1_ASM2085478v1_genomic	80.38	25.65	23.48	76.38
GCF_041305605.1_ASM4130560v1_genomic	79.36	36.05	35.86	92.7
GCF_002067275.1_ASM206727v1_genomic	78.95	34.24	31.96	92.7
GCF_036802725.1_ASM3680272v1_genomic	78.92	34.49	33.88	95.92
GCA_002508425.1_ASM250842v1_genomic	78.35	27.4	30.06	95.26
Methanomethylovorans hollandica DSM 15978	78.25	26.34	31.79	99.84
GCF_963678545.1_piPlaSpea1.Methanomethylovorans_sp_1.1_genomic	78.07	20.62	30.53	99.84
GCF_041447285.1_ASM4144728v1_genomic	77.02	24.16	30.56	99.84
GCA_001896725.1_ASM189672v1_genomic	76.52	24.47	23.74	99.18

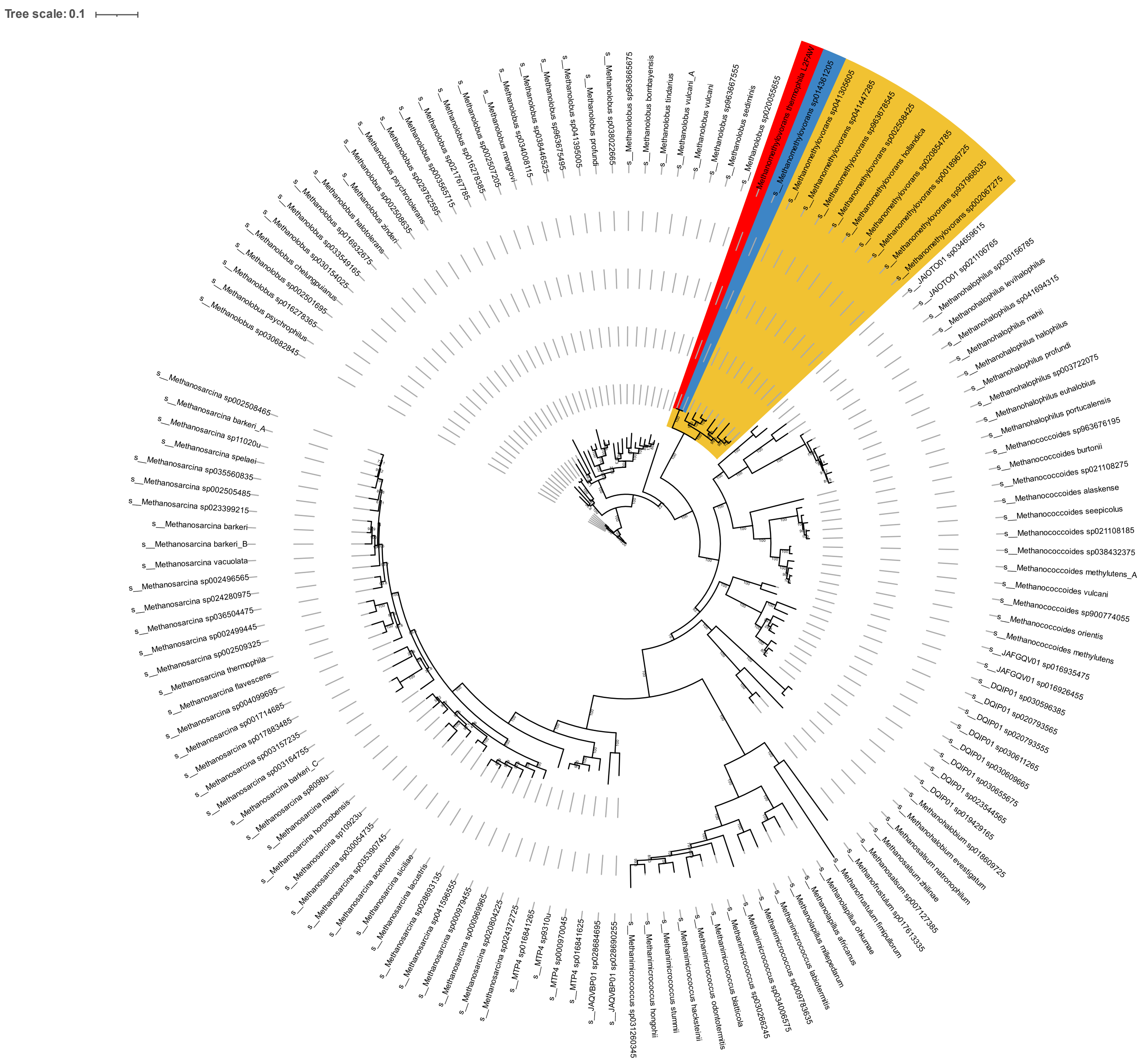

Figure S1: Phylogenomic tree based on the 53 archaeal marker proteins of all available GTDB species representatives (n=139) and our assembled genome Methanomethylovorans thermophila L2FAW. In red our assembled genome, in blue the MAG with the highest similarity to our genome (ANI=99.9%), and in yellow all members of the genus Methanomethylovorans. Given numbers at the branches reflect bootstrap values.
